# Complement Cascades and Brain Disorders

**DOI:** 10.3390/biom15081179

**Published:** 2025-08-17

**Authors:** Ivana Jovčevska, Alja Videtič Paska, Katarina Kouter

**Affiliations:** 1 Institute of Biochemistry and Molecular Genetics, Faculty of Medicine, University of Ljubljana, 1000 Ljubljana, Slovenia; ivana.jovcevska@mf.uni-lj.si (I.J.);; 2 Institute of Microbiology and Immunology, Faculty of Medicine, University of Ljubljana, 1000 Ljubljana, Slovenia

**Keywords:** complement system, immune system, humoral immunity, innate immunity, psychiatric disorders, neurodegeneration, neurology, oncology

## Abstract

The complement system is a vital component of innate immunity. Besides its roles in pathogen defense, its significance in neurodevelopment, neurodegeneration, and cancer progression is beginning to be recognized. We performed a comprehensive literature review to summarize the involvement and dysregulation of the complement system in three main CNS-associated conditions: Alzheimer’s disease, schizophrenia, and glioma. In Alzheimer’s disease, activation of the complement system contributes to neuroinflammation, synaptic loss, and neuronal death. In glioblastoma, complement promotes tumor growth, immune evasion, and therapy resistance. In schizophrenia, genetic variations in complement components, particularly C4A, are associated with synaptic pruning abnormalities and disease susceptibility. We conclude that the complement system has a dual role of protector and pathogenic mediator in the central nervous system. While it is critical in neurodegenerative, oncological, and psychiatric disorders, its role is not understood well enough. For therapeutic purposes, targeting the complement system may open new frontiers for therapeutic interventions without disrupting important physiological processes. More research is needed to elucidate the exact roles of the complement and help translate these findings into clinical settings.

## 1. Introduction

The complement system is a complex part of the innate immune system. This intricate cascade comprises over 50 components of both soluble and membrane bound components [1]. First described by the name complement in the late 19th century by Paul Ehrlich [2], it was long believed to be a heat-labile component and a complementary mechanism to antibodies in the defense against pathogens, hence the name “complement”. Now we know that the complement system is much more than that and that it has a number of roles both in the functioning of the healthy organism and in the development of pathologies including autoimmune disease [3], kidney pathologies [4], oncology [5], neurology [6], and others. All of these pathologies represent a major burden worldwide, with estimates of the prevalence of autoimmune diseases in the industrialized world population reaching up to 10% [7]. Chronic kidney disease, although often overlooked, also affects up to 10% of the world′s population and contributes significantly to quality of life and mortality [8]. In both oncology and neurodegeneration, the global burden is significant and rapidly increasing. Studies estimate that there were almost 20 million new cancer diagnoses in 2022 alone. Statistically, one in five people will be diagnosed with cancer in their lifetime, and cancer will be the cause of death in around one in ten people [9]. Diseases associated with the central nervous system (CNS) are one of the main determinants of disability-adjusted life years (DALYs), where DALYs are an indicator of a combined burden including both mortality and morbidity [10]. Furthermore, it is estimated that the global burden of brain disorders will increase by 22% by 2050, equating to a staggering 4.9 billion cases worldwide [11].

**Figure 1:**
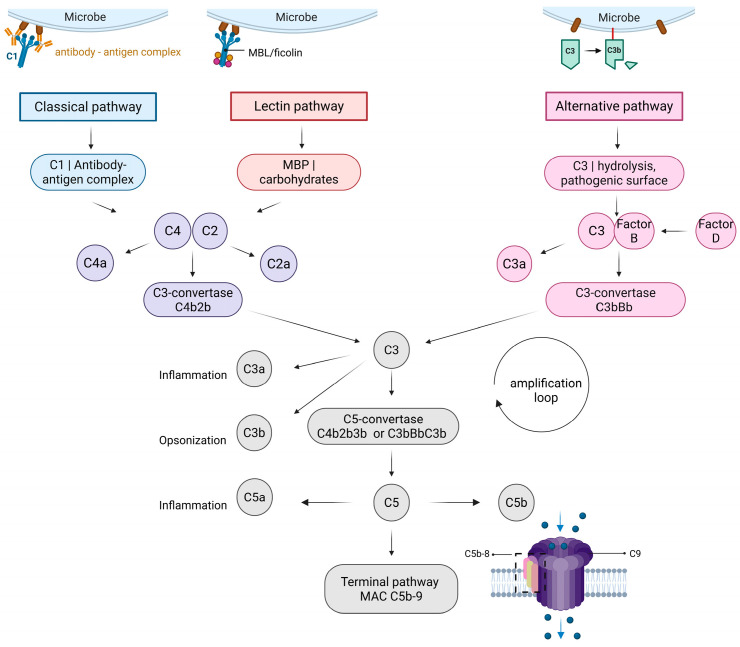
The complement cascade. The complement cascade is divided into three pathways: the classical, the lectin, and the alternative pathways. The classical pathway is activated by the binding of C1q to immune complexes. The lectin pathway is activated by the binding of pattern recognition molecules (PRMs) to carbohydrate surfaces. The alternative pathway can be triggered by C3 hydrolysis or foreign surfaces. Different complexes can form after a cascade reaction. The classical and lectin pathways form identical C3- and C5-convertase complexes. The alternative pathway forms a different variant of C3- and C5-convertase, but the function remains similar. The formation of C5-convertase can lead to the terminal pathway that is responsible for the formation of the membrane attack complex. MBP—mannose-binding protein. Created in https://BioRender.com. Jovčevska, I. (2025) https://BioRender.com/suucx9w (accessed on 25 July 2025).

The complement system functions as a control system of natural immunity. Perhaps its most well-known role is in pathogen defense. The complement cascade ends with the formation of a pore-forming complex that efficiently destroys foreign pathogenic cells. During complement cascade activation, surface deposition of complement components also leads to opsonization and phagocytosis. Additionally, the complement cascade is involved in immune cell recruitment, inflammation, and activation of platelets and epithelial cells. Complement is also involved in tissue homeostasis maintenance, serving as a cell waste disposal and immune clearance system [1]. Another important function of the complement system is its involvement in synaptic pruning, the process of removing the inactive or less active synapses as a form of fine-tuning of the brain circuit [12]. The complement cascade can be activated via three pathways: the classical, lectin, and alternative pathways (see Figure 1). The main distinctions between the three pathways are the source of activation and the mechanism of C3- and C5-convertase formation.

### 1.1. Classical Pathway

The classical pathway is triggered by complexes of antigens and antibodies that form on pathogens or other surfaces. These complexes are recognized by C1q, which binds with the Fc region of the antibody–antigen complex, and it can bind directly to the surface as well. Once bound, conformational changes allow for the activation of C1r, a serine protease that cleaves and activates C1s, leading to cleavage of C4 into two fragments, C4a and C4b. C4b fragments, still bound to the surface, can bind C2 (which is later cleaved by C1s as well) into two parts: C2a and C2b [6]. In recent years, there was a nomenclature change regarding the two parts, as the names were switched. Looking at the complement cascade, component cleavage fragments marked with “a” are smaller and do not bind with other complement cascade components, while larger component cleavage fragments marked with “b” do. The only exception is C2, where the larger cleavage fragment, originally named C2a, binds to C4b to form a C3-convertase. To unify the nomenclature, an initiative was started to switch the names of the cleavage fragments C2a and C2b. Readers should therefore be mindful when reading the older literature, as some sources will describe the larger fragment of C2 by the old name, C2a [13]. By binding C4 and C2 fragments, C3-convertase is formed (historically named C4b2a, more recently renamed C4b2b). The role of C3-convertase is the cleavage of C3 into C3a and C3b. C3b fragments can bind to C3-convertase, forming a C5-convertase (C4b2b3b), which further cleaves C5 into C5a and C5b [14].

### 1.2. Lectin Pathway

The lectin pathway was the last complement pathway to be discovered. It resembles the classical pathway, as both share a larger part of the cascade, with the same complexes of C3- and C5-convertase [15]. The main difference between the classic and lectin pathways is the activation point. The lectin pathway is triggered by pattern recognition molecules (PRMs) such as mannose-binding lectin (MBL), ficolins, and collectins that recognize carbohydrates on foreign surfaces of pathogens or changed host cells. Next, surface-bound PRMs form complexes with MBL-associated serine proteases, MASP [16]. In the first step, MASP-1 autoactivates, which leads to activation of the second MASP, MASP-2. The PRM-MASP-1 complex can cleave C2, while PRM-MASP-2 is able to cleave both C4 and C2, which forms the previously mentioned classic/lectin C3-convertase C4b2b and further follows the classical pathway [15].

Roughly 5 to 30% of the population shows signs of lectin pathway deficiency. This is mostly due to genetic variations of MBL, but other components of the lectin pathway can be involved as well. The clinical picture can vary greatly, making some of those who are deficient more prone to infection, while the majority of those who are deficient present no clinical symptoms [17].

### 1.3. Alternative Pathway

From an evolutionary point of view, the alternative pathway appears to be the oldest of the three pathways and is not dependent on antibodies or carbohydrates. The alternative pathway shows low levels of consistent spontaneous activation by hydrolysis of C3 to C3(H_2_O), working as a monitor surveillance system in the body (also known as the “tick over theory”) [18]. The alternative pathway can also be activated by foreign surfaces, which have bound C3b fragments that can also act as opsonins, marking it for phagocytosis.

C3(H_2_O) and C3b can bind factor B, a plasma protein. Factor D, a serine protease also known as C3 proactivator convertase, cleaves factor B into fragments Ba and Bb [19]. Bb remains bound, thus creating C3(H_2_O)Bb (this is called fluid phase C3-convertase), which further cleaves C3 into C3a and C3b. Fragment Bb can join with fragments of C3b, creating a C3bBb, another form of C3-convertase, the alternative pathway C3-convertase. This convertase is not very stable by itself, so it needs to be stabilized by properdin, which strengthens protein–protein interactions and allows for further cleavage of C3 into C3a and C3b. C3b fragments can join the C3-convertase, creating a C5-convertase, C3bBbC3b [20].

Another characteristic of the alternative pathway is its ability to quickly generate large amounts of C3b through the complement amplification loop, serving as a fast response mechanism. All three complement pathways can generate C3b, which can attach to the cell surface and can potentially form new C3-convertases that produce new C3b fragments, driving the amplification loop [21].

### 1.4. Terminal Pathway

All three previously described pathways of the complement system converge at the terminal lytic pathway. C5-convertase (whether in the form of alternative C3bBb3b or classic/lectin C4b2b3b) cleaves C5 into C5a and C5b, initiating the terminal pathway [22]. C5a is released and can serve as a chemoattractant and anaphylatoxin, while C5b binds with complement components, forming the terminal complement complex, also known as the membrane attack complex (MAC) or C5b-9. More specifically, C5b first binds rapidly to C6 (C5b-6 complex) and C7 (C5b-6-7 complex), after which the complex binds to the outer part of the plasma membrane. Next, C8 is bound, forming the C5b-6-7-8 complex. The binding of components creates conformational changes that allow the complex to be partially inserted into the membrane. Lastly, multiple components of C9 join, forming a pore in the membrane that causes the lysis of the cell [23]. MAC is important in defense against pathogens such as bacteria, so people with genetic deficits of components are more exposed to infections, especially by *Neisseria meningitidis* and *Streptococcus pneumoniae* [24].

### 1.5. Regulation of the Complement System

The complement system needs to be tightly regulated to ensure its appropriate and efficient activation. When overly activated or activated against inappropriate triggers (such as biomaterials or post-transplantation), the activation can work against an individual’s own cells [25]. Similarly, complement deficiency and underactivation can lead to damage from ineffective clearance (for example, SLE) and the development of chronic autoimmune or inflammatory diseases [26]. Therefore, it is crucial that there are mechanisms that keep complement under control with precise and tight regulation. The main inhibitors of the classical and lectin pathways are C1 inhibitor, factor I, and C4b binding protein (C4BP). The main inhibitors of the alternative pathway are factor H and factor I [27].

In the classical pathway, C1 inhibitor inhibits C1s and C1r, while in the lectin pathway, it regulates active MASP. C1 inhibitor also inhibits the contact system proteases FXIIa, FXIa, and kallikrein of the kinin pathway, a plasma system associated with inflammation, vasodilation, and pain [15].

Factor I is a common inhibitor of all three complement pathways. Factor I is a serine protease that can inactivate C3b and C4b in the presence of cofactors such as factor H, C4BP, membrane cofactor protein (MCP), and complement receptor 1 (CR1). Factor H is an important regulator of the alternative pathway, binding to C3b (in fluid phase or on the cell surface) and preventing the formation of C3-convertase by inactivating C3b as a cofactor to factor I [28]. Multiple options are available for the deactivation of C4b. Bound C4b (as well as C3b) can be inactivated with the help of CR1 and/or MCP, followed by factor I [20]. C4BP is a fluid-phase inhibitor of the classical and lectin pathways. Shaped like an octopus, it can bind multiple C4b molecules at the same time, either in the fluid phase or bound to the cell surface. This can dissociate the C3-convertase and can serve as a cofactor for FI in the cleavage of C4b [29].

Another common inhibitor of the complement pathway is membrane protein decay-accelerating factor (DAF), which works by preventing the formation and accelerating the decay of the C3-convertase complexes (both classical and alternative) [15].

## 2. Complement and the CNS

### 2.1. Production of Complement Components in the CNS

**Figure 2:**
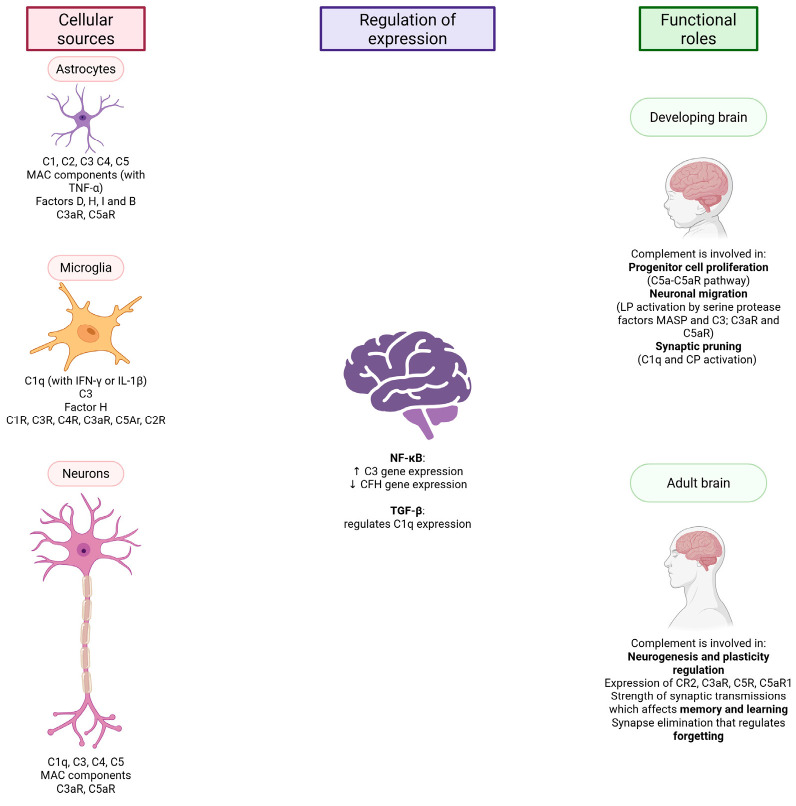
A schematic representation of the hidden underlying mechanisms and expression regulation factors of the complement in the CNS. LP—lectin pathway; CP—classical pathway. Created in BioRender. Jovčevska, I. (2025) https://BioRender.com/v18o059 (accessed on 25 July 2025).

Most blood complement components are produced by hepatocytes; the concentration of complement proteins increases as part of the acute phase response. But complement components can be produced by other cells as well, e.g., adipocytes produce factor D (adipsin), myeloid cells properdin, and lymphoid cells produce various complement components [30]. Individual components are also synthesized in the brain, as summarized in Figure 2. The main sources of complement component production are astrocytes and microglia. Astrocytes express the components C1, C2 [31], C3, C4, C5, MAC components (in the presence of TNF-α) [32], factor D [31], factor H, factor I, and factor B, as well as the receptors C3aR and C5aR. Microglia express C1q (in the presence of interferon-γ (IFN-γ) or IL-1β), C3, factor H, the receptors C1R, C3R, C4R, C3aR and C5Ar [32] and, according to some sources, also C2R [31]. Expression of C1q, C3, C4, C5, MAC components and the receptors C3aR and C5aR has been observed in neurons [31]. The regulation of complement synthesis and activity in the CNS is mainly mediated by inflammatory nuclear factor κ B (NF-κB), acting as a transcription factor increasing C3 gene expression and decreasing CFH gene expression, and TGF-β, which regulates C1q expression in neurons. Studies show that complement components are expressed in the brain, but not all, not everywhere, and not in the same proportions. Based on this, we can conclude that complement in the brain also performs functions that do not involve the full functioning of the entire cascade [32].

### 2.2. The Complement System and the Developing Brain

The components of the complement system are more highly expressed during pre- and postnatal development of the CNS, whereas their expression decreases in the adult brain. The role of the complement system during brain development can be divided into three categories: (i) progenitor cell proliferation (C5a-C5aR pathway), (ii) neuronal migration (the subset of the lectin pathway involving activation by the serine protease factors MASP and C3; C3aR and C5aR) and (iii) synapse pruning (C1q and the classical activation pathway). Inappropriate progression of these processes can lead to neurological and mental developmental disorders, e.g., schizophrenia and autism spectrum disorders [32]. The key role of the components of the complement system in the development of a healthy brain and the maintenance of homeostasis has been demonstrated in many studies on animal models [33].

Synaptic pruning is an important process for brain maturation and neurocircuit refinements in the early developmental stage. During this process, the excess of synapses is eliminated. Synapses that are weak or less active are removed in a process mediated by microglial cells. Such synapses are tagged with C1q, followed by involvement of C3 and microglial C3R [12]. C1q synthesis can be increased by astrocytes, probably by signaling with TGF-β [34]. The exact process that ensures an appropriate number and strength of synapses is not yet clear, but studies show that more C1q is localized at presynaptic terminals, where apoptosis-like processes occur [35].

### 2.3. The Complement System and the Adult Brain

While complement is important during brain development, research is increasingly indicating that the complement system plays an important part in adulthood as well. Complement is responsible for the regulation of neurogenesis in the adult mammalian brain, thus affecting its plasticity. Components are produced in the adult brain under normal physiological conditions, as well as in pathological states. Animal models, as well as human postmortem studies, have proven the expression of complement components in adulthood. Neural stem cells (responsible for neurons that develop later in life) and immature neurons express the complement receptors CR2, C3aR, C5R, and C5aR1. In addition, complement together with microglia regulate the strength of synaptic transmissions and thus affect memory and learning [31]. Complement is also crucial for forgetting, an important process mediated by synapse elimination [36]. In a study by Wang et al. (2020), mice that were overexpressing a complement inhibitor DAF showed increased levels of freezing (less forgetting) and a higher reactivation rate between cells compared to control mice that did not overexpress DAF. This further indicates the importance of complement activity in forgetting [37].

## 3. The Complement System and CNS Pathologies

Traditionally, the CNS has been described as immune-privileged. However, recent research has shown that adaptive and innate immune responses can be elicited in the CNS under normal development and pathological conditions [38]. The complement system is a bridge between the innate and adaptive immune systems [39]. Changes in the complement activation in the brain, either underregulation or overactivation, can lead to abnormal tissue development, e.g., synaptic remodeling or synapse loss associated with neurodegenerative and psychiatric disorders [40]. Furthermore, the complement system can promote carcinogenesis (sustain proliferative signaling, angiogenesis, and resistance to apoptosis), activate migration and invasion, and modulate anti-tumor activity [41]. Complement proteins can be found in the brain in conditions that disrupt the integrity of the blood–brain barrier (BBB) [38]. Neoplastic diseases, however, can modulate the complement proteins in order to achieve a pro-tumorigenic microenvironment. In glioblastoma, for example, the immune environment contributes to therapy resistance, and besides escaping immune surveillance, glioma cells are involved in immune suppressive activities to promote malignant transformation [42].

Although complement testing is not yet fully integrated into routine testing and standard clinical practice for CNS disorders, there is increasing evidence of its potential diagnostic, prognostic, and mechanistic importance. Table 1 summarizes the most important complement-related biomarkers identified in Alzheimer′s disease, schizophrenia, and glioblastoma. It provides an overview of the results of several studies that highlight both common and distinct complement changes in these conditions. In the following sections, we will explore these findings in more detail and discuss their biological implications and significance for disease progression and therapeutic targeting.

Research on Alzheimer′s disease, schizophrenia, and glioblastoma suggests altered levels or genetic variation of complement components, emphasizing the common involvement of the complement system in CNS pathology. It can be noted (see Table 1) that changes in C1q and C3 are present in all three disease states, suggesting a common role in neuroinflammation and immunomodulation. In both Alzheimer′s disease and schizophrenia, increased levels of components of the classical complement pathway (C1q, C4) are associated with disease progression, which could indicate excessive synaptic pruning or chronic inflammation. Glioblastoma in particular shows a distinct pattern with a likely tumor-specific upregulation of C3a/C3aR and downregulation of factors such as FB and C1r in the blood, suggesting a role in promoting an immune environment that is supportive of the tumor.

In all three conditions, dysregulation of the complement system may be a contributing factor. The consequences of this dysregulation, whether neurodegeneration, inadequate neurological development, or immune evasion, are specific to each disease state and may be shaped by the underlying pathology and immune context.

### 3.1. Alzheimer’s Disease

The risk of neurodegeneration increases dramatically with age. As the world population’s age is gradually rising, predictions estimate that by 2050, there will be 113 million people affected by dementia. With approximately 55 million people living with Alzheimer’s disease (AD) worldwide, it is the most common form of neurodegenerative disease and the most common cause of dementia. This neurodegenerative state is characterized by the presence of extracellular plaques (containing amyloid β) and intracellular neurofibrillary tangles (containing tau) [54]. The severity of AD manifestation varies from patient to patient, ranging from no observed impairment to more commonly observed mild cognitive impairment, gradually progressing to dementia as the most severe final stage [54]. The origin of the disease can be sporadic or familial. The majority of cases appear to be sporadic (late onset), while the familial type (early onset, in up to 5% of cases) is seen in subjects under the age of 65 and is observed as an underlying autosomal dominant presentation of AD-associated genes [55]. While in sporadic (late onset) cases of AD, genetic influences are not so clearly defined, genetics does play a role in it as well. As AD is a complex multifactorial disease, increasingly research is also directed towards studying various epigenetic mechanisms and their association with AD [56].

Currently, there are numerous hypotheses on the cause and progression of AD. The complement system could be involved in more than one hypothesis, including the amyloid hypothesis and the neuroimmunology hypothesis.

The commonly accepted amyloid hypothesis presents amyloid β as the main cause of AD. Incorrect processing of the amyloid precursor protein (APP) could lead to amyloid β plaque formation. In addition to the protein itself, the cellular and extracellular environment could also be important, as they can create different biophysical conditions. Studies have investigated the importance of ionic strength and the presence of divalent cations such as calcium. For example, S100A9 and α-synuclein, proteins that are both involved in amyloid formation and associated with neurodegeneration, show a dependence on the chemical environment. S100A9 is able to form oligomers in the absence of calcium, but the mechanical properties are markedly different when calcium is present, indicating the modulating effects of calcium [57]. Similarly, ionic strength can control the nature of fibril formation in the case of alpha-synuclein. This effect also appears to be influenced by protein concentration. These findings suggest that biophysical conditions in the brain may influence amyloid aggregation, which can modulate both neuroinflammation and the tendency for complement activation. Although these proteins are not directly associated with complement activation in the cited studies, they can influence the emergence of an inflammatory-prone environment that may contribute significantly to activation of the complement system [58].

Under normal conditions, excess amyloid β is removed via glial cell activation and complement system-mediated phagocytosis, indicating that the complement system can have a protective role under normal conditions. This changes under neuroinflammatory conditions, as the amyloid clearance is flawed, further resulting in the presence of proinflammatory cytokines and complement components [21]. Complement activation can therefore contribute to neuroinflammation, which can lead to synapse dysfunction, synapse loss, and the death of neuronal cells, all observed in AD.

One of the early studies of the complement system and AD was done by Rogers et al. in 1992. Looking at brain samples of AD patients, they observed colocalization of C1q in AD pathological structures compared to the control group tissue. Additional in vitro assays also confirmed that amyloid β can activate the complement classical pathway [59]. Soon, many studies followed, confirming the involvement of C1q, C3, and C4 in AD [44]. C3 and C1q also play an important role in glial cells. Glial cells are important in AD pathology, as they serve as effector cells, releasing signaling molecules [60]. In AD, both activated microglia and astrocytes are involved. Classically activated M1 microglia (which are proinflammatory) can secrete IL-1α, C1q, and TNF. Secretion of such signal molecules can aid in the formation of A1 reactive astrocytes, which have a great capacity for causing neuronal death and synaptic loss. A1 astrocytes have been observed in AD and show a very high increase in C3 expression [61]. When looking at plasma exosomes derived from the astrocytes in AD patients, there was an increase in the complement components C1q, C4b, C3d, factor B, factor D, Bb, C3b, and C5b-C9, but not MBL, which indicates the involvement of the classical and alternative pathways. This further suggests that type A1 reactive astrocytes could be a generator of proinflammatory mediators, with exosomes as a way of cargo distribution between cells [43]. Levels of C3, C4, and CR1 complement receptor 1 have also been investigated in the cerebrospinal fluid (CSF). When comparing samples of AD patients and a control group of mild cognitive impairment, increased levels of C3 and C4 were observed in the CSF of AD patients [45].

Finally, it appears that there is an additional genetic component of the complement, correlating the changes in gene expression of complement genes with AD pathology. Studies have looked into CR1, which binds opsonized particles/pathogens via C3b/C4b, regulates complement activity, and appears to be associated with the amyloid β metabolism and accumulation/clearance process [46]. A systematic review by Lu et al. evaluated various polymorphisms of CR1 as risk factors for late-onset AD. Looking at two CR1 polymorphisms, rs3818361 and rs6656401, they observed significant allele frequency differences between late-onset patients and the control group [47]. Similar results were observed in a meta-analysis of 18 studies, which confirmed that rs3818361 and rs6656401 represent increased risk factors for late-onset AD in Caucasians [48].

### 3.2. Schizophrenia

Schizophrenia is a chronic disorder that belongs to the most serious psychiatric illnesses, with a lifetime prevalence of ~1% [62]. It develops in early adulthood [62], and can be characterized by positive and negative symptoms, such as delusions, hallucinations, disorganized speech, catatonic behavior, decreased ability to express emotions (affective flattening), and reduced interest in social activities [63]. Through neuroimaging methods, gray matter loss [64] and a decrease in dendritic spine density [65] have been associated with schizophrenia. The exact mechanism for these pathological changes is not clear, but it could be associated with erroneous synapse pruning and elimination. In the past few decades, the disorder has been linked to systemic inflammation and the immune system, where the role of the complement system as an immune mediator could be significant. Even though the etiology of schizophrenia is not fully elucidated, there is enough evidence to consider the heritability factors that could significantly contribute to the disorder burden. Epidemiological studies showed that heritability represents one of the risk factors [66]. Twin [67] and adoption [68] studies further supported this idea and provided an estimate of the genetic component of around 80%. Among psychiatric disorders, schizophrenia is the most studied in association with complement genetic variants, gene expression, and protein function. So far, the results for the studies seem to be rather inconsistent and rarely replicated.

The most comprehensive search for genetic variants in schizophrenia is the hypothesis-free study performed by the Schizophrenia Working Group of the Psychiatric Genomics Consortium [69] and the International Schizophrenia Consortium [70]. In their genome-wide association study (GWAS), the major histocompatibility complex (MHC) extended region has been recognized as a schizophrenia risk locus. The MHC loci lie near the C4 gene of the complement system [69]. In the study of Sekar et al., the variety of different C4 alleles showed an influence on C4 gene expression levels, resulting in diverse C4A and C4B expression in the brain. The allelic variations were associated with schizophrenia through their ability to increase the expression of C4A [49]. Higher expression of C4A was further associated with the presence and severity of delusions, which could be linked to the complement role in synaptic pruning [71]. Currently, we cannot argue whether these variants specifically contribute to the disorder or determine how they interact with the environment and potentially modify the disorder; therefore, further functional studies should try to discern these contributions.

Among the complement proteins, the most studied in schizophrenia are the ones comprising the classical pathway, C1q, C2, C3, and C4. In the case of C1q, it was determined that significantly elevated levels in the serum of mothers during pregnancy were a risk factor for schizophrenia and psychosis development in their children. These results imply that measurement of C1q in the prenatal period could be a screening tool for detrimental immune system activation [50]. Elevated levels of C1q were also reported in drug-naïve, first-episode schizophrenia patients, together with increased levels of C3, worse memory recall, higher values of C4 and thinner sensory cortex [51]. A more comprehensive study showed further positive correlation for body mass index and C-reactive protein levels in the serum with C3 and C4 markers [72].

Data on the lectin and alternative pathways are very limited. In more recent studies, complement lectin pathway alterations were associated with schizophrenia. Significantly higher levels of plasma MASP-2 were determined in female patients compared to controls, and were also associated with a type of schizophrenia. The correlation was further observed for alternative pathway functional activity and MASP-2 level. Additionally, the L-ficolin-MASP-2 complex that activates the lectin cascade showed correlation with downstream C2 component hemolytic activity, while L-ficolin itself correlated with classical pathway functional activity and C-reactive protein serum concentrations [73].

### 3.3. Glioblastoma

A grade IV malignant glioma, glioblastoma, is one of the most common, aggressive, and lethal cancers in adults worldwide. Even with aggressive treatment consisting of surgery, chemotherapy, and radiation, patients’ life expectancy is approximately 15 months [74]. One of the reasons for the poor disease outcome is the ability of glioblastoma cells to inactivate the body’s immune response. The role of the complement system in glioblastoma has not been extensively studied, as can be seen by the few publications available. Förnvik et al. examined the role of complement inactivation in glioblastoma in silico, in vitro, and in vivo [75]. First, they analyzed a previously published data set (27k data) from in-house generated cDNA microarrays and reported complement 1 inactivator (C1-IA) overexpression in tissue samples from 26 glioblastoma patients compared to non-malignant controls, i.e., four epilepsy patient samples. The second in silico analysis used data from the publicly available database GSE4290, Affymetrix Gene Chip Analysis [76], consisting of 23 non-tumor brain samples and 77 glioblastoma samples, and showed a 3.2-fold change of C1-IA in glioblastoma compared to controls. The authors confirmed this finding in vitro at the gene expression level and detected a 2.9-fold change of C1-IA in glioblastoma compared to epilepsy controls. Their IHC analysis confirmed C1-IA overexpression in glioblastoma. Finally, using a Fischer 344 rat model intracranially inoculated with GFP-expressing NS1 glioblastoma cells, the authors examined whether reactivation of the complement system could affect the survival of rats. They injected GFP-positive NS1 cells coated with rabbit anti-human C1-IA or no antibodies (controls). They detected increased survival of animals injected with cells coated with antibodies compared to the controls (29.7 ± 4.0 days versus 23.0 ± 3.4 days, respectively). The authors concluded that inhibiting C1-IA may be beneficial for glioblastoma patients.

In a different study, Ah-Pine et al. investigated the role of C3a and its receptor C3aR in various primary and secondary brain tumors [52]. The authors found that glioblastomas contain high numbers of tumor-associated macrophages (TAMs) that express C3bR and vascular endothelial growth factor (VEGF). Fewer such cells were found in grade II and III gliomas. With IHC, they showed abundant C3aR expression in grade IV diffuse gliomas, while in non-tumor brain tissue, C3aR cells were scarce. C3aR was not found in perivascular mesenchymal or endothelial cells. Moreover, C3a/C3aR expression was evaluated using U251MG glioblastoma cells and THP1 macrophages. THP1 cells expressed higher gene expression levels of C3, C3aR, Cathepsin L, VEGF, and CCL5/RANTES when compared to U251MG. Specifically, C3aR was 10–20-fold more expressed in THP1 than in U251MG. C3aR glioblastoma TAMs are thought to drive angiogenesis and metastasis and may also contribute to immunosuppression and promote tumor growth. The authors concluded that their results confirmed that the glioblastoma tumor microenvironment may favor increased expression of C3a and C3aR.

Related to this, Lim et al. showed that mesenchymal stem-like cells (MSLCs) boost glioma invasion through the C5a/p38/ZEB1 axis [77]. MSLCs are similar to mesenchymal stem cells (MSCs) but differ in their surface antigens. However, MSLCs alone did not form tumors in mice. The authors cocultured glioblastoma cells and MSLCs to determine if they have a tumorigenic role in brain tumors. The invasiveness of glioblastoma cells was increased when they were cocultured with MSLCs. In vivo injection of glioblastoma cells coinjected with MSLCs was able to infiltrate adjacent regions to a greater extent than injected glioblastoma cells alone. Mice injected with glioblastoma cells and MSLCs developed tumors faster and presented with poorer survival than those injected with glioblastoma cells alone or with bone marrow–derived MSCs (BM-MSCs). The authors analyzed soluble factors and found increased levels of C5a, GROα, IL-6, and IL-8 in the glioblastoma–MSLCs coculture. Of these, only C5, the precursor of C5a, was increased in the MSLCs cells alone. Their results showed that MSLCs secrete C5a, which increases expression of ZEB1 by activating p38 MAPK in glioblastoma cells. In this way, MSLCs enhance the invasion of glioblastoma cells into brain parenchymal tissue.

Soluble factor H (FH) regulates the alternative pathway by acting as a cofactor for factor I-mediated cleavage of C3b, competes with factor B to bind to C3b, and promotes the dissociation of C3 convertase, C3bBb [42]. FHR5 is the largest member of the FH family and can bind C3b, heparin, C-reactive protein, and iC3b (the cleavage product of C3b). DeCordova et al. showed that cultured primary tumor cells isolated from glioblastoma patients secrete functionally active FHR5, which regulates the complement by acting as a cofactor for factor I-mediated cleavage of C3b, and degrading (decaying) C3 convertase. In the culture supernatant, secreted FHR5 was detected with a western blot band corresponding to 65 kDa. Over a 48 h growth period, ELISA showed that the amount of secreted FHR5 increased with the growing number of glioblastoma cells, i.e., more primary glioblastoma cells = greater quantity of secreted FHR5. Using in vitro assays, the authors proved that the secreted FHR5 was biologically active by using the hemolytic assay to show that primary glioblastoma cell-secreted FHR5 exhibited dose-dependent inhibition of sheep erythrocyte lysis. In addition, they showed that FHR5 enabled factor I to cleave C3b into its inactive fragment iC3b and was able to decay C3 convertase dose dependently.

Serum levels of key proteins of the complement system have also been related to glioblastoma. Bouwens et al. studied the degree and activation pathways of complement activation in tissue and serum samples of patients with different stages of glial tumors [41]. Quantitative differences in the three complement pathways, classical, alternative, and lectin, were detected with ELISA for C1q, MBL, and factor B on patient and control serum samples. The authors observed increased levels of C1b (145 ± 4 μg/mL versus 121 ± 2 μg/mL) and MBL (870 ± 75 ng/mL versus 404 ± 41 ng/mL), and decreased levels of factor B (1556 ± 100 aU/mL versus 1996 ± 85 aU/mL) in patients compared to controls. To detect MBL, C1q, and factor B in tissue sections, they performed IHC. Here, C1b was strongly present in the tumor tissue, while MBL and factor B were also present but showed weak staining compared to C1b. Moreover, C3, which is the common point of the three initiating pathways of the complement system, was abundantly present in necrotic and non-necrotic areas of glioblastomas. Finally, in glioblastoma, the authors detected the C5b-9 complex in individual glioblastoma cells, which suggests that up to this point, complement activation had taken place locally.

Similarly, Förnvik Jonsson et al. examined complement components in the peripheral blood of adult patients with IDHwt glioblastoma [53]. They tested samples from 40 patients, but due to limitations in sample volume, some of the analyses were not performed in all samples. C1r was reduced in 17 out of 28 patients, and C1q was reduced in 17 out of 39 patients. The function of the classical pathway was analyzed in 16 cases by determining the amounts of C3d (elevated in 2 cases), C1q, and C1r (reduced in 5 cases). The coagulation factors INR-PK and APTT were reduced in 2 and 28 patients, respectively, while platelet counts were reduced in 1 and increased in 2 patients. Finally, the authors found a correlation between reduced levels of both C1r and C1q and worse patient survival. However, due to a small sample size, this finding needs to be further confirmed.

Besides being expressed in a variety of tumor types, the complement system also mediates the interactions within the tumor microenvironment. Complement-related molecules such as C1q, C3a/C3aR, and C5a/C5aR have been found in tumor microenvironments [78]. So far, results of the published studies implicate the involvement of innate immunity in the pathophysiology of glioblastoma. However, the precise involvement of the complement cascade in the pathophysiology of glioblastoma remains to be understood. This can be achieved by in vivo studies to reveal the precise influence of complement activation in glial tumors.

## 4. Potential Clinical Use

### 4.1. Complement and Laboratory Diagnostics

Timely and appropriate complement testing is an important translational and clinical aspect of complement disorders. With the increasing awareness of the importance of the complement system, laboratory diagnosis of complement is becoming an increasingly important area. There is currently a wide range of tests available (see Table 2 for the most commonly used tests). The clinical interpretation of complement diagnostics is complex and requires careful selection of tests (usually starting with functional evaluation of complement activity and then, based on the results, adding appropriate tests). Special attention needs to be paid to patients who have a current infection (possible increased activation of the complement system) or who have undergone a process such as plasma exchange [79]. An additional caveat in complement testing is the importance of sample handling, from patient sample collection to the beginning of the testing procedure. Complement can activate ex vivo due to the increased external temperature and freeze/thaw cycles [80]. Laboratories, therefore, need to prepare their own sample rejection criteria and sample handling protocols.

The increasing use of anti-complement therapeutics (discussed in more detail later in this chapter) may further complicate the interpretation of complement diagnostics. It is of great importance that when ordering tests, clinicians disclose the possible use of anti-complement therapies; otherwise, false test claims may be made. One such example is C3 inhibitors. If they are used as complement inhibitors, the C3 level in the blood can be significantly increased [81].

### 4.2. Complement and Therapeutics

Currently, much research is focused on the use of complement in therapeutics as summarized in Table 3. So far, anticomplement therapy has had great success, notably in the treatment of paroxysmal nocturnal hemoglobinuria and complement-related kidney pathology. The most-used anticomplement therapeutics are eculizumab (anti-C5 monoclonal antibody), ravalizumab (anti-C5 monoclonal antibody with a longer half-life), pegcetacoplan (C3 inhibitor), and iptacopan (factor B inhibitor). Additional biologicals acting on the activation pathways are being explored, with sutimlimab (anti-C1s, authorized for use in the EU) and narsoplimab (anti-MASP-2) leading in development [82].

The main challenge of anticomplement therapy stems from the complexity and the importance of the complement cascade. By blocking one part of the cascade, the functionality of the complement is disrupted. While this might benefit some conditions of the patient, it can also be a disadvantage. For example, patients prescribed eculizumab have an increased risk of meningococcal infections, while patients receiving pegcetacoplan have a greater risk of *Neisseria meningitidis*, *Streptococcus pneumoniae*, and *Hemophilus influenzae type B* infections. Before the start of the anticomplement treatment, patients are advised to complete the vaccination process and use antibiotic prophylactic treatment, with additional monitoring from the clinician [83].

The use of anticomplement treatment in the CNS has been limited so far. Eculizumab is starting to be used in the treatment of neuromyelitis optica spectrum disorder (NMOSD). NMOSD is characterized by the inflammation of the CNS, often affecting the optic nerve and the spinal cord. Aquaporin 4 (AQP4) is the most common water channel protein in the CNS. Patients often present with AQP4 autoantibodies, which can trigger the complement [84].

The current treatment options in AD are very limited, with even fewer therapeutic options including complement [21]. Some studies, however, do investigate AD and complement. One therapy option is anti-amyloid beta antibodies, such as lecanemab, which has been approved by the FDA [86]. Hettmann et al. (2020) designed a variation of the anti-amyloid beta antibody that does not activate the complement system. This antibody, while bound to amyloid beta, inhibits C1q binding and prevents the response by microglia [90]. Other studies tested PMX205, a C5a receptor 1 antagonist. In a mouse model of AD treated with PMX205, Gomez-Arboledas et al. (2022) observed a reduced amyloid load, synaptic loss, and dystrophic neurites, while promoting a neuroprotective microglial phenotype. These results are promising, as a C5a receptor 1 antagonist CCX168 (Avacopan) is already in use for the treatment of anti-neutrophil cytoplasmic autoantibody-associated (ANCA) vasculitis [87].

To our knowledge, there are currently no studies on mental disorders like schizophrenia and complement-targeted therapies, but complement could be useful in clinical settings. A recently published study used proteome profiling of extracellular vesicles (EVs) and machine learning as a method for schizophrenia diagnosis and treatment response prediction. In patients with schizophrenia, significantly more complement protein was observed in EVs (namely C3, C4A, C4B, C4BPA, and C4BPB). Further, these EV-based biomarkers were able to distinguish schizophrenia from bipolar disorder and major depressive disorder [91]. In clinical settings, several proinflammatory biomarkers proved to be elevated in schizophrenia naïve patient serum [92]. These data again imply that other proteins involved in the immune response and inflammation, like the complement system, should be further studied as potential players in the development and treatment of the disorder.

With glioblastoma being one of the most aggressive and lethal types of cancer, new treatment opportunities are gravely needed. There are currently not many studies examining the potential of the complement system as a therapeutic option. An animal study, using rats inoculated with glioma cells, compared the efficiency of radiotherapy and anti-C1-inhibitor antibodies on the survival of the animals. Animals with either subcutaneous or intracranial tumors were treated either with radiotherapy, anti-C1-inhibitor, or a combination of both. In the subcutaneous tumor group, the combined treatment prolonged survival. In the intracranial tumor group, anti-C1-inhibitor treatment had no effect on survival; the authors concluded that the doses of anti-C1-inhibitor were not enough to achieve any survival effect [89]. This study highlights an additional problem of therapeutic interventions in the CNS—the problem of delivery and achieving/and or sustaining the therapeutic concentration [85].

## 5. Conclusions

The complement system is emerging as an important player in the pathophysiology of different CNS disorders such as Alzheimer’s disease, schizophrenia, and glioma. As described, the complement has diverse roles, ranging from synaptic pruning to neurodegeneration and tumor progression. Even with significant advances, there is still much that is unknown in the field of complement, both in terms of how the complement system itself works and the relevance of this information for clinical use. However, the increasing knowledge of the link between complement and various physiological and pathological conditions promises new possibilities for the use of the complement system in clinical practice. Future research should focus on the development of targeted strategies to inhibit the pathological activation of the complement without disrupting its physiological functions. This way, novel diagnostic and therapeutic avenues addressing unmet needs in neurodegenerative, oncological, and psychiatric disorders can be opened.

## Figures and Tables

**Table 1:** Complement-related biomarkers in Alzheimer’s disease, schizophrenia, and glioblastoma.

Disease	Complement-Related Biomarker	Potential Diagnostic or Prognostic Value	Sample	
Alzheimer’s disease	C1q, C3, C4	Increased levels linked to disease progression	Blood, CFS, brain	[43,44,45]
	CR1	Genetic variations associated with increased risk for late-onset AD	Blood	[46,47,48]
Schizophrenia	C4	Genetic variations associated with increased risk	Blood	[49]
	C1q, C2, C3, C4	Increased levels linked to disease progression	Blood	[50,51]
Glioblastoma	C3a/C3aR	Increased expression linked to poor prognosis	Tumor	[52]
	C3	Increased levels linked to poor prognosis	Tumor	[41]
	C1b, MBL	Increased levels associated with increased risk	Blood	[41]
	FB	Decreased levels associated with increased risk	Blood	[41]
	C1r, C1q	Decreased levels linked to poor patient survival	Blood	[53]

AD—Alzheimer’s disease; CFS—cerebrospinal fluid; CR1—complement receptor 1; FB—factor B; MBL—mannose-binding lectin.

**Table 2:** Summary of most commonly used tests in complement diagnostics.

Testing Type	Sample	Component	Consideration	Methodology
Functional testing	Serum	Classical pathway Alternative pathway Lectin pathway	To look for complement deficiency and assess activation state of the pathways	Hemolytic assays, EIA
Individual component quantification and functional assessment	EDTA-plasma/serum	C1, MBL, ficolins 1–3, properdin, C2–C9, FB, FD	Component may be present but in a dysfunctional state Monitoring of anti-complement therapy	Radial immunodiffusion, nephelometry, EIA
Control protein	EDTA-plasma/serum	C1-inhibitor, C4BP, FI, FH, properdin	To investigate suspected imbalance of complement	Immunodiffusion, nephelometry, EIA
Activation products	EDTA-plasma	C3a, C3dg, C4a, C4d, Bb, C5a, sC5b9	To distinguish primary from secondary complement deficiency To assess the involvement of an individual pathway To investigate suspected imbalance of complement	Rocket immunoelectrophoresis, EIA
Autoantibodies	EDTA-plasma/serum	anti-C1q, anti-C1-INH, anti-MBL, anti-C3b, anti-FH, anti-FI, anti-FB, C3-Nefs	Detected in autoimmune diseases	EIA, hemolytic assays
Molecular analysis of complement genes	EDTA whole blood	Genes of interest	To confirm complement disorder	NGS. hydrolysis probes

C4BP—C4b binding protein; EDTA—ethylenediaminetetraacetic acid; EIA—enzyme immunoassay; FB—factor B; FD—factor D; FH–factor H; FI—factor I; MBL—mannose-binding lectin; NGS—next generation sequencing.

**Table 3:** Summary of most common anticomplement therapeutics.

Therapeutic Agent	Target	Disease	Advantages	Limitations	Reference
Eculizumab	C5	PNH aHUS gMG NMOSD	Approved by the FDA and EMA Safe Well-tolerated	2000-fold increased risk of meningococcal infections Focused on rare diseases Burden on healthcare system Limited accessibility	[82,83,84,85]
Ravalizumab (Ultomiris))	C5	PNH	Approved by FDA Longer half-life than eculizumab Improved dosing frequency	Target diversity results in not reaching clinical endpoints	[82,84,85]
Pegcetacoplan (Empaveli, Apellis)	C3	PNH	Approved by the FDA and EMA Superior to eculizumab Long-term treatment is well tolerated	Greater risk of *Neisseria meningitidis*, *Streptococcus pneumoniae*, and *Hemophilus influenzae type B* infections	[82,85]
Lecanemab (Leqembi)	Aβ	AD	Approved by the FDA Able to slow patients’ cognitive decline in early disease stages Highly selective for Aβ protofibrils Lowers Aβ plaques Minimizes Aβ deposition Improves clinical degradation	Amyloid-associated imaging defects (edema or microhemorrhages)	[86]
PMX205	C5aR1	AD	Reduces total number of Aβ plaques and total Aβ load, number of ThioS + plaques, synaptic loss, and dystrophic neurites Promotes neuroprotective microglial phenotype	Might have an effect in the initial plaque deposition stages, but not once the amyloid plaques are formed	[87]
CCX168 (Avacopan, Chemocentryx/Vifor)	C5aR1	ANCA-associated vasculitis	Already in use Oral administration	10% of patients developed renal and urinary disorders	[88]
Anti-C1 inhibitor antibody	C1	Glioma (rat model)	Potential radiosensitizing effect, i.e., increased survival of animals with subcutaneous glioblastoma	Drug delivery issues Difficult to achieve and/or sustain therapeutic concentration	[89]

PNH—paroxysmal nocturnal hemoglobinuria; aHUS—atypical hemolytic uremic syndrome; gMG—generalized myasthenia gravis; NMOSD—neuromyelitis optica spectrum disorder; FDA—Food and Drug Administration; EMA—European Medicines Agency; Aβ—amyloid-beta; AD—Alzheimer’s disease; C5aR1—C5a receptor 1; ANCA—anti-neutrophil cytoplasmic autoantibody.

## Data Availability

No new data were created or analyzed in this study. Data sharing is not applicable to this article.

## Abbreviations

The following abbreviations are used in this manuscript:

Aβ	Amyloid-beta
AD	Alzheimer’s disease
aHUS	Atypical hemolytic uremic syndrome
ANCA	Anti-neutrophil cytoplasmic autoantibody
APP	Amyloid precursor protein
APTT	Activated partial thromboplastin time
AQP4	Aquaporin 4
BBB	Blood–brain barrier
BM-MSCs	Bone marrow–derived MSCs
C1-IA	Complement 1 inactivator
C4BP	C4b binding protein
C5aR1	C5a receptor 1
CFH	Complement Factor H
CNS	Central nervous system
CR1	Complement receptor 1
CSF	Cerebrospinal fluid
DAF	Decay-accelerating factor
DALYs	Disability-adjusted life years
EDTA	Ethylenediaminetetraacetic acid
EMA	European Medicines Agency
EIA	Enzyme immuno-assay
EVs	Extracellular vesicles
FDA	Food and Drug Administration
FB	Factor B
FD	Factor D
FH	Soluble factor H
FI	Factor I
FHR5	Complement factor H–related protein 5
GFP	Green fluorescent protein
gMG	Generalized myasthenia gravis
GWAS	Genome-wide association study
IDHwt	Isocitrate dehydrogenase wild type
IFN-γ	Interferon-γ
IHC	Immunohistochemistry
MAC	Membrane attack complex
MASP	MBL-associated serine proteases
MBL	Mannose-binding lectin
MCP	Membrane cofactor protein
MHC	Major histocompatibility complex
MSCs	Mesenchymal stem cells
MSLCs	Mesenchymal stem-like cells
NF-κB	Nuclear factor κ B
NGS	Next generation sequencing
NMOSD	Neuromyelitis optica spectrum disorder
PNH	Paroxysmal nocturnal hemoglobinuria
PRMs	Pattern recognition molecules
SLE	Systemic lupus erythematosus
TAMs	Tumor-associated macrophages
TGF-β	Transforming growth factor beta
TNF-α	Tumor necrosis factor
VEGF	Vascular endothelial growth factor
